# Comparative evaluation of the Minimally-Invasive Karyotyping (MINK) algorithm for non-invasive prenatal testing

**DOI:** 10.1371/journal.pone.0171882

**Published:** 2017-03-17

**Authors:** Tianjiao Chu, Patricia A. Shaw, Suveyda Yeniterzi, Mary Dunkel, Aleksander Rajkovic, W. Allen Hogge, Kimberly D. Bunce, David G. Peters

**Affiliations:** 1 Department of Obstetrics, Gynecology and Reproductive Sciences, University of Pittsburgh, Pennsylvania, United States of America; 2 Center for Fetal Medicine, Magee-Womens Research Institute, Pittsburgh, PA, United States of America; Hospital Authority, CHINA

## Abstract

Minimally Invasive Karyotyping (MINK) was communicated in 2009 as a novel method for the non-invasive detection of fetal copy number anomalies in maternal plasma DNA. The original manuscript illustrated the potential of MINK using a model system in which fragmented genomic DNA obtained from a trisomy 21 male individual was mixed with that of his karyotypically normal mother at dilutions representing fetal fractions found in maternal plasma. Although it has been previously shown that MINK is able to non-invasively detect fetal microdeletions, its utility for aneuploidy detection in maternal plasma has not previously been demonstrated. The current study illustrates the ability of MINK to detect common aneuploidy in early gestation, compares its performance to other published third party methods (and related software packages) for prenatal aneuploidy detection and evaluates the performance of these methods across a range of sequencing read inputs. Plasma samples were obtained from 416 pregnant women between gestational weeks 8.1 and 34.4. Shotgun DNA sequencing was performed and data analyzed using MINK RAPIDR and WISECONDOR. MINK performed with greater accuracy than RAPIDR and WISECONDOR, correctly identifying 60 out of 61 true trisomy cases, and reporting only one false positive in 355 normal pregnancies. Significantly, MINK achieved accurate detection of trisomy 21 using just 2 million aligned input reads, whereas WISECONDOR required 6 million reads and RAPIDR did not achieve complete accuracy at any read input tested. In conclusion, we demonstrate that MINK provides an analysis pipeline for the detection of fetal aneuploidy in samples of maternal plasma DNA.

## Introduction

Over recent years there has been rapid development and clinical deployment of non-invasive methods for the detection of fetal aneuploidy[1, 2]. One method that has gained particular prominence is the genome-wide shotgun sequencing of maternal plasma DNA. Proof of concept for this was first demonstrated in October 2008[3] and the resulting field of non-invasive prenatal testing (NIPT) has since evolved rapidly (reviewed in [1, 4]).

The utility of the MINK approach was originally demonstrated using a model system in which the genomic DNA of a trisomy 21 male individual was mixed with the genomic DNA of his mother at dilutions of 5% and 10%[5]. MINK has since been used to non-invasively detect the presence of a fetal microdeletion[6, 7]. Although NIPT for aneuploidy is now well established clinically[1], the demonstration that MINK is effective in a clinical context is significant because it utilizes a unique statistical model that may impact both the accuracy and the economics of clinical aneuploidy testing.

In the current study, we evaluated the utility of MINK for the analysis of shotgun sequencing data generated from maternal plasma DNA samples collected in early gestation from women carrying normal and abnormal fetuses. These data were also analyzed by two recently published third party methods known as RAPIDR[8] and WISECONDOR[9]. Finally, we sought to determine the minimum number of sequencing reads that are required by each method for accurate aneuploidy detection.

## Materials and methods

### Human DNA samples

The University of Pittsburgh Institutional Review Board approved the patient consenting process and collection of all samples used in this study. Written informed consent was obtained in every case. Patient characteristics are summarized in Table 1. Individuals meeting the inclusion criteria were consecutively recruited and samples collected as described below.

**Table 1:** Distribution of the size of the plasma libraries.

	Minimum	Median	Maximum
Total Reads	1,797,079	22,499,423	98,598,829
Aligned Reads	858,511	14,979,604	73,274,600

Total Reads: Number of all reads in a library.

Aligned Reads: Number of reads in a library that are aligned uniquely and without mismatch to human reference genome GRCh37.

#### Inclusion criteria

The inclusion criteria for this study were as follows. Participants were pregnant women who underwent prenatal genetic counseling within the Center for Medical Genetics at Magee-Womens Hospital. Women were referred for genetic counseling for a variety of reasons, including desire to have prenatal diagnostic procedures such as amniocentesis or chorionic villus sampling, advanced maternal age (>35), abnormal first trimester serum screen and nuchal translucency test, previous history of a fetus or child with an autosomal or sex chromosome aneuploidy, structural anomalies identified by ultrasonography, parental carrier of chromosome inversion or translocation, or parental aneuploidy or mosaicism for aneuploidy. Only women who underwent genetic counseling within the Center for Medical Genetics were included.

#### Exclusion criteria

Women who were not pregnant or those who did not wish to participate were excluded. Children (under 18 years of age) were also excluded. This is because the relative rarity of eligibility in children, as compared to adults, made their inclusion impractical and unnecessary. Each participant’s DNA was coded in a manner that the subject cannot be identified, directly or through identifiers linked to the subject.

### Plasma DNA extraction and sequencing

Plasma was separated from whole blood via centrifugation at 1,600 x g for 10 minutes, followed by a second centrifugation to remove contaminating nucleated cells at 16,000 x g for 10 minutes. DNA was extracted from plasma using the QIAamp DNA Blood Mini kit (Qiagen, Valencia, CA). Plasma DNA libraries were prepared using standard Illumina TruSeq protocols (Illumina, San Diego, CA) or NEBNext Ultra Library Prep Kit for Illumina (New England Biolabs, Ipswich, MA). Libraries were quantified via real time PCR and via bioanalyzer (Agilent) analysis and sequenced on either a HiSeq 2000 or 2500 (Illumina) using 50bp single-end reads. Reads were aligned to the human reference genome GRCh37 using Bowtie and duplicates were removed. Each chromosome was divided into non-overlapping 50kb regions. The number of reads that aligned uniquely and exactly to each region was counted. Loess regression was applied to the read counts against the average GC content of each region to reduce GC bias. Statistical analysis of data was performed using the MINK algorithm[5].

### MINK algorithm

The MINK algorithm, as implemented in this study and previously reported (1), regresses the log2 ratios of the read counts of diploid chromosomes between a test library and each of the normal reference libraries against the GC content of these. The fitted models then are used to predict the log2 ratios for the target chromosome where the test library is suspected to be aneuploidy, between the test library and each of the normal reference libraries. The t statistics and the associated p values are calculated based the differences between the observed and predicted log2 ratios for the target chromosome. The test library is considered aneuploidy if the median of all p values < = 0.05.

### Calculation of fetal fraction

Fetal fraction was determined using quantitative real time PCR analysis of the SRY and HBB genes. Primers and probe sequences for the real time PCR reaction were obtained from Maron, et al (Maron, 2007), and are as follows:

*SRY*: Forward primer 5’–TCCTCAAAAGAAACCGTGCAT-3’

Reverse primer 5’–AGATTAATGGTTGCTAAGGACTGGAT- 3’

Probe– 5’- FAM- CACCAGCAGTAACTCCCCACAACCTCTTT-TAMRA-3’

*B-globin*: Forward primer 5’-GTGCACCTGACTCCTGAGGAGA-3’

Reverse primer– 5’-CCTTGATACCAACCTGCCCAG-3’

Probe– 5’-FAM-AAGGTGAACGTGGATGAAGTTGGTGG-TAMRA-3’

Bglobin is a ubiquitous housekeeping gene and was run concurrently with the SRY to ensure that DNA was present for each sample, irrespective of fetal gender. In order to estimate DNA concentration in the plasma DNA, standard curve DNA was run simultaneously with the plasma DNA. The standard curve DNA was prepared using commercially available DNA with known concentration. The range of values for the standard curve was 6.4pg/5ul to 20,000pg/5ul. For each real time PCR reaction, 12.5ul 2x TaqMan Universal PCR Master Mix, 1.25ul 10uM forward primer, 1.25ul 10uM reverse primer and 0.0625ul 100uM probe were combined. 10ul plasma DNA, 5ul standards or 10ul water (to serve as negative control) were added to the appropriate wells. Each plasma DNA sample and the negative control were run in triplicate. The standard curve DNA was run in duplicate. The thermal cycling conditions were an initial denaturation step of 95oC for 10 minutes, followed by 50 cycles of 95oC for 15 sec and 60oC for 1 min. The real time PCR reactions were performed using the 7900HT Sequence Detection System (Applied Biosystems).

### Selection of sequence reads for titration experiments

40 karyotypically normal libraries, split evenly between males and female fetuses, were chosen to serve as the reference set for MINK, WISECONDOR, and RADPIDR downsample testing. Uniquely aligned read counts in these libraries ranged from 10.3 to 39.7 million reads. Libraries tested were 10 karyotypically normal libraries (all male fetuses) and 10 trisomy 21 libraries (4 female, 6 males). The test libraries were each downsampled using Picard (http://broadinstitute.github.io/picard/) with default settings to 20, 15, 10, 8, 6, 4, 2 and 1 million reads. The reference libraries were not downsampled. Third party software packages were used in their default modes. 3 settings for RAPIDR testing were used: GC correction only, GC correction with PCA, and NCV. The WISECONDOR results reflect the Windowed Aneuploidy Test output.

## Results

Clinical characteristics for all patients in the study are shown in S1 Table. Plasma DNA samples were subjected to single end sequencing for 50 cycles on an Illumina HiSeq 2000 or 2500. Summary statistics for sequencing data are shown in Table 1. All samples were successfully sequenced and data interpreted. Sequencing data files are available on request from the corresponding author.We collected a total of 416 plasma samples and generated 447 libraries (some samples were sequenced multiple times). Karyotypes of these samples are summarized in Table 2. All normal and abnormal karyotypes were confirmed by traditional karyotyping methods performed via amniocentesis or chorionic villus sampling or, in a minority of cases, post partum.

**Table 2:** Karyotypes of plasma samples and sequencing libraries. Note that the number of libraries is greater than the number of samples, because some samples were sequenced multiple times.

	Libraries	Plasma Samples
Total	447	416
Normal	377	355
Trisomy 13	2	2
Trisomy 14	1	1
Trisomy 18	11	10
Trisomy 21	56	48

We arbitrarily selected 234 normal libraries as reference and then tested each of the 416 samples (447 libraries) against each of the 234 reference libraries for chromosomes 13, 14, 18, and 21 using the MINK algorithm. (If a library is itself a reference library, it was tested against a subset of the reference libraries not including itself.) S1–S4 Figs show the box plots of the p values of the MINK tests for the 61 trisomy samples (70 libraries) against the reference libraries for chromosomes 13, 14, 18, and 21 respectively. S5–S8 Figs show the p values of the MINK tests for the 355 normal samples (377 libraries) against the reference libraries for chromosomes 13, 14, 18, and 21 respectively. In each plot, a library is colored in red if it was generated from a sample trisomic for the corresponding chromosome, and blue otherwise. A library is considered to be trisomic for a given chromosome if the median of the p values is less than or equal to 0.05. It is considered normal if the median p value is greater than or equal to 0.1. If the median p value is between 0.05 and 0.1, it is considered ambiguous and needs further investigation. As shown in Table 3, the MINK algorithm performed extremely well achieving high sensitivity and specificity for trisomies 21, 18 and 13.

**Table 3:** Performance of the MINK algorithm for Trisomy 13, 18, and 21.

	Estimate	conf.int1	conf.int2
T21.SEN	0.9791	0.8753	0.9989
T18.SEN	1.0000	0.6555	1.0000
T13.SEN	1.0000	0.1979	1.0000
All.SEN	0.9836	0.9002	0.9991
T21.SPC	1.0000	0.9871	1.0000
T18.SPC	1.0000	0.9883	1.0000
T13.SPC	0.9976	0.9845	0.9999
All.SPC	0.9994	0.9960	1.0000
T21.PPV	1.0000	0.9059	1.0000
T18.PPV	1.0000	0.6555	1.0000
T13.PPV	0.6667	0.1253	0.9823
All.PPV	0.9836	0.9002	0.9991

Rows

SEN: Sensitivity of the MINK algorithm for the given Trisomy.

SPC: Specificity of the MINK algorithm for the given Trisomy.

PPV: Positive predictive value of the MINK algorithm for the given Trisomy.

All.SEN/All.SPC/All.PPV: Sensitivity/Specificity/Positive predictive value of the MINK algorithm for the combined test of Trisomy 13, 14, 18, and 21.

Columns

Estimate: Estimated sensitivity/specificity/positive predictive value.

conf.int1: Lower bound of the 95% confidence interval

conf.int1: Upper bound of the 95% confidence interval

One apparently false positive trisomy 13 case (PL1909) was identified (S1B Fig, two libraries—PL1909A and PL1909B—were generated from this sample). Given that we have previously identified a case of partial maternal mosaicism for trisomy 21[10] we sequenced pure samples of maternal and amniocyte genomic DNA corresponding to PL1909 and analyzed the resulting data using MINK. Both samples were entirely normal with no evidence of maternal or fetal aneuploidy (data now shown). We also identified a single false negative case for trisomy 21 (PL789) (S1 Fig). Because low levels of fetal DNA in maternal plasma can theoretically account for false negative findings, we determined fetal fraction for this sample by quantitative PCR for SRY sequence and found it to be ~7%, which is likely to be well within the acceptable range (data not shown). To further investigate this pregnancy, pure maternal genomic DNA was purified, sequenced and analyzed for copy number anomalies using MINK. These results were entirely normal with no evidence of any anomaly that might explain the false negative data.

In order to evaluate MINK in context against other methods, we evaluated two other analysis pipelines by testing their performance using the same data sample set described above. The combined results of these analyses are summarized in Table 4. It can be seen that Wisecondor called more false negatives than MINK and demonstrated lower sensitivity, especially for chromosome 18. RapidR called more false positives than MINK and demonstrated lower specificity. Moreover, the libraries where MINK reported inaccurate results (PL1909 for chromosome 13, PL789 for chromosome 21) are also incorrectly reported by both Wisecondor and RapidR. This suggests that either these two libraries are extremely hard to test, or that the karyotype information about them is incorrect. Note that, using the two samples proportion test, MINK’s false positive rate was found to be significantly lower than that of RapidR (p value = 0.0092). However, because of sample size, the difference in false negative rate reported by MINK versus Wisecondor is not statistically significant (p value = 0.6111).

**Table 4:** Performance comparison between MINK, WISECONDOR and RAPIDR.

Chromosome	MINK (False Positive)	RAPIDR (False Positive)	WISECONDOR (False Positive)	MINK (False Negative)	RAPIDR (False Negative)	WISECONDOR (False Negative)
chr13	1	1	1	0	0	0
chr18	0	5	0	0	0	1
chr21	0	5	0	1	1	2

**Fig 1:**
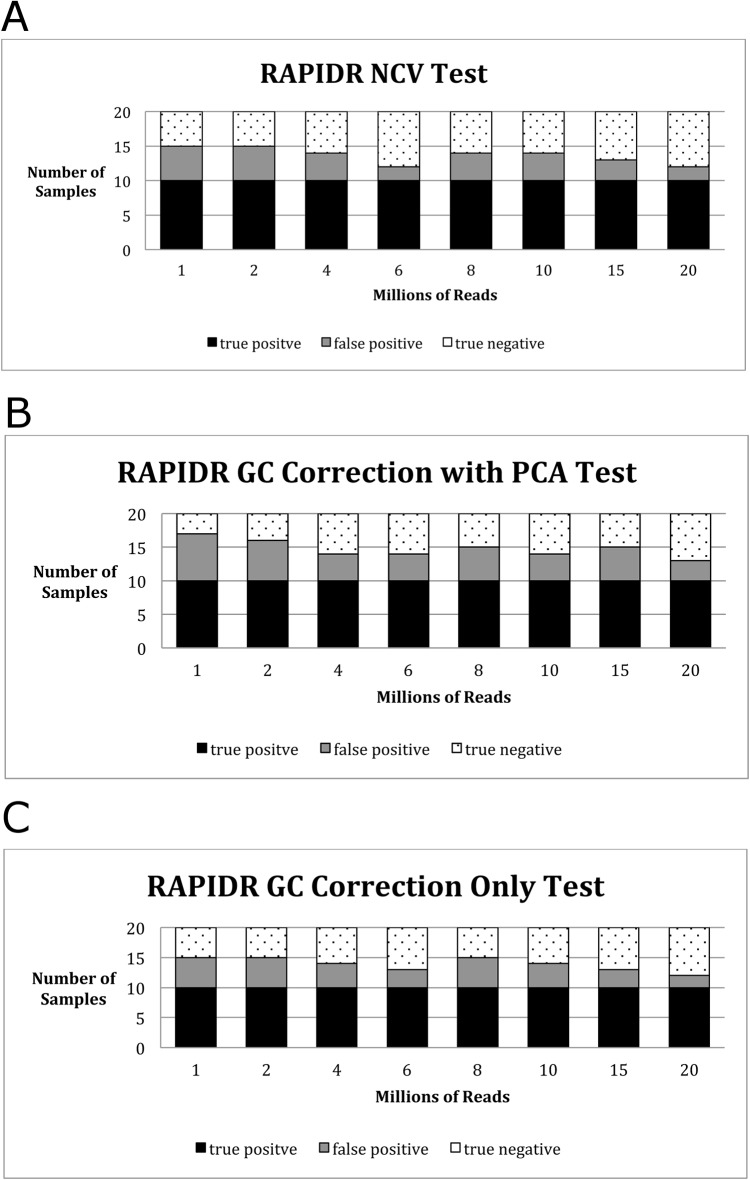
**(A)** Sensitivity of RAPIDR NVC test for Trisomy 21 detection across a range of input read counts. **(B)** Sensitivity of RAPIDR GC correction with PCA test for Trisomy 21 detection across a range of input read counts. **(C)** Sensitivity of RAPIDR GC correction only test for Trisomy 21 detection across a range of input read counts.

**Fig 2:**
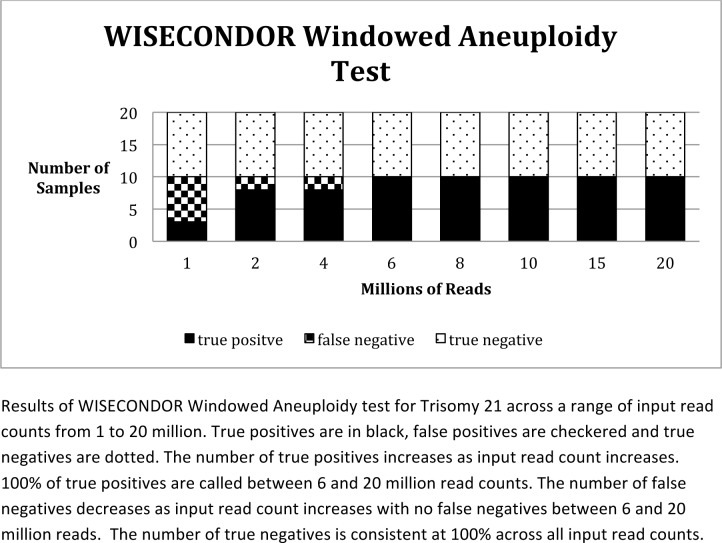
Sensitivity of WISECONDOR for Trisomy 21 detection across a range of input read counts.

**Fig 3:**
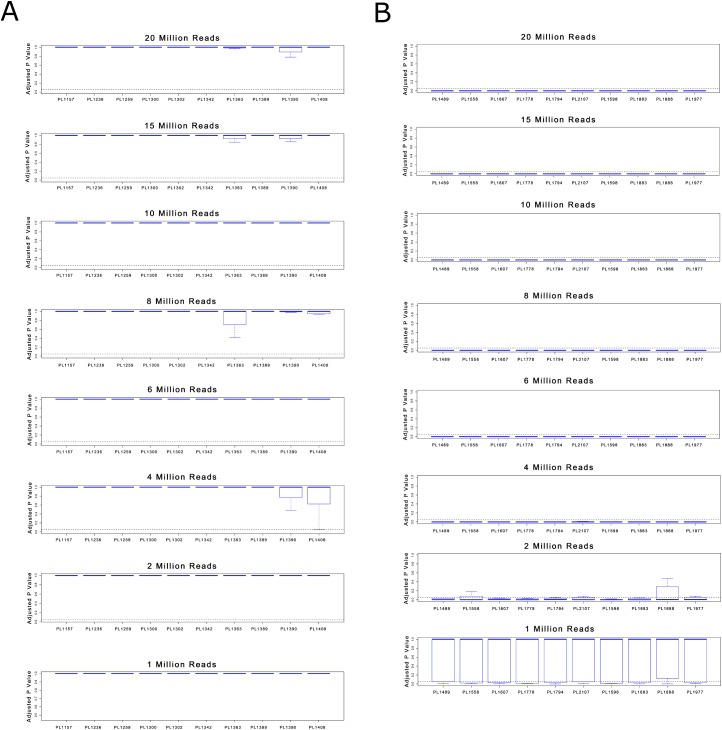
**(A)** Sensitivity of MINK for euploidy detection across a range of input read counts. **(B)** Sensitivity of MINK for Trisomy 21 detection across a range of input read counts.

Finally, we sought to determine the relationship between the number of input reads and the sensitivity of MINK, WISECONDOR and RAPIDR. This was achieved by randomly sampling sequence reads from each data set using the DownsampleSAM tool in Picard (http://broadinstitute.github.io/picard/). We selected inputs of 1, 2, 4, 6, 8, 10, 15 and 20 million input reads and analyzed these using WISECONDOR and RAPIDR. As shown in Fig 1 and Table 5, under all 3 conditions and across all read inputs, RAPIDR had numerous false positive results. Under all 3 conditions (RAPIDR NCV Test, GC Correction with PCA Test and GC Correction Only Test), RAPIDR was able to detect all true positives for each read input. However, at all input levels, it failed to report 100% of the true negatives. No false positives were found at any read input level reported by WISECONDOR (Fig 2 and Table 5). WISECONDOR was able to detect all true negatives at each level and was able to detect all true positives down to a level of 6 million reads. Below 6 million reads, WISECONDOR generated false negative results (Fig 2 and Table 5). We then undertook the same analyses with the same input data using MINK. As show in in Fig 3 and Table 5, when input library size was reduced to 1 million, we found a false negative rate of 100%. But at 2 million or above, the false negative rate was 0%. Furthermore, the false positive rate was found to be 0% for the normal samples at all read inputs. Only trisomy 21 was tested due to low case numbers for trisomy 13 and 18 in our samples.

**Table 5:** Summary of read down sampling experiment.

Millions of Reads (Input)		MINK	WISECONDOR Windowed Aneuploidy Test	RAPIDR NCV	RAPIDR GC Correction plus PCA	RAPIDR GC Correction
20	True Positive	10	10	10	10	10
	True Negative	10	10	8	7	8
	False Positive	0	0	2	3	2
	False Negative	0	0	0	0	0
15	True Positive	10	10	10	10	10
	True Negative	10	10	7	5	7
	False Positive	0	0	3	5	3
	False Negative	0	0	0	0	0
10	True Positive	10	10	10	10	10
	True Negative	10	10	6	6	6
	False Positive	0	0	4	4	4
	False Negative	0	0	0	0	0
8	True Positive	10	10	10	10	10
	True Negative	10	10	6	5	5
	False Positive	0	0	4	5	5
	False Negative	0	0	0	0	0
6	True Positive	10	10	10	10	10
	True Negative	10	10	8	6	7
	False Positive	0	0	2	4	3
	False Negative	0	0	0	0	0
4	True Positive	10	8	10	10	10
	True Negative	10	10	6	6	6
	False Positive	0	0	4	4	4
	False Negative	0	2	0	0	0
2	True Positive	10	8	10	10	10
	True Negative	10	10	5	4	5
	False Positive	0	0	5	6	5
	False Negative	0	2	0	0	0
1	True Positive	0	3	10	10	10
	True Negative	10	10	5	3	5
	False Positive	0	0	5	7	5
	False Negative	10	7	0	0	0

## Discussion

MINK performed effectively for the accurate early gestational detection of fetal aneuploidy in a set of 416 maternal plasma samples. Using the same sample set, MINK outperformed two published third party methods (Wisecondor and RapidR) by demonstrating superior sensitivity and specificity. Furthermore, when the input requirements of all three methods was evaluated by downsampling the number of input reads, MINK was able to effectively return accurate results even at 2 million read inputs, which is significantly fewer than that required by these alternative methods.

One possible explanation for the superior performance of MINK is that it employs a sophisticated statistical model that provides a representation of the whole genome sequencing data using a linear statistical model for read counts derived from each chromosome (or large regions of genome). MINK adopts a two-step statistical test procedure. In the first step, the linear statistical model is fit using tag count data for the chromosomes that are believed to be diploid from both a normal control library and the library to be tested. In the second step, the fitted model is applied to the observed tag count data for the target chromosome in the two libraries, and the p value of the observed data against the null hypothesis that the target fetal chromosome in the library to be tested is diploid is calculated. This model explicitly includes the interaction between experimental specific factors and the chromosome specific factors. For example, information about the DNA samples and libraries, such as the median length of DNA fragments, number of PCR cycles, can be used as covariates in the model. This enables MINK to effectively remove or reduce the bias introduced by these factors, hence achieving higher power.

A number of other approaches published prior to MINK[3, 11] did not consider such factors. Specifically, in the Z score method published by Chiu *et al*[11] the standard deviation and the mean of the percentage of tags coming from the target chromosome in a set of normal libraries is calculated, and the target chromosome in a test library is considered to be trisomic if the observed value in the new library is more than 3 standard deviations from the mean of the normal samples. Similarly, Fan *et al*[3] uses a Student’s t test to test if the tag density for the target chromosome in the new library is different from the tag density for the target chromosome in a group of normal libraries. The majority of algorithms published after MINK have taken only an ad-hoc approach by pre-processing the data using, for example, a GC correction approach[12]. Details of algorithms employed in the commercial setting were not available for comparison.

Furthermore, MINK makes full use of the sequencing data from the whole genome, not only the target chromosome, and can be used to test a new library when only a single normal library is used as the control. MINK fits the target library to the statistical model by using the information from karyotypically normal genomic regions of the target library, and uses the fitted model to predict the expected counts of the region of interest (e.g., chromosome 21). In contrast, other algorithms predict the region of interest in the target library by only the size (or median coverage) of the whole library and the targeted chromosome, and essentially assuming all the libraries are otherwise the same except for the targeted chromosomes[3, 8, 11–13]

Additionally, MINK allows the calculation of the power of the statistical test when combined with a reliable estimate of the percentage of fetal DNA, hence greatly facilitating the decision making process which requires both the p value and the power of the statistical test.

Of note are the identification of a single false positive and a single false negative case. The false negative case did not appear to be the consequence of low fetal fraction since this was determined by quantitative PCR and found to be adequate. It is of course possible that these findings are the result of disconcordant placental and fetal karyotypes, secondary to placental mosaicism. Specifically, for the false positive case this could involve the presence of aneuploid placental cells despite a normal fetal karyotype. Unfortunately we were not able to confirm this because the patient elected to undergo amniocentesis rather than CVS. Therefore the DNA available to us was fetal in origin and had no utility for placental karyoptying. Similarly, the false negative case could be the result of the opposite form of disconcordance, reflecting a karyotypically normal placenta despite an aneuploid fetus. Unfortunately we were unable to obtain CVS from this pregnancy to test this hypothesis.

Given that NIPT for aneuploidy is emerging as a new clinical standard of care, it is important that it is delivered in the most economical way possible. With this in mind, the finding that MINK is accurate at low read input is significant. his is because the generation of sequencing reads is a major component of the cost of NIPT. Although other fixed costs will not be affected by the ability to analyze fewer sequencing reads (for example, DNA extraction and library preparation), the ability to detect aneuploidy with fewer sequence reads reduces the cost of cycle sequencing and allows an increase in the number of samples that can be multiplexed together on a given sequencer. It also increases flexibility by enabling the use of sequencers with lower throughput (eg MiSeq versus HiSeq).

In summary, we have extended our original communication of the MINK method[5] by demonstrating that MINK has utility for the detection of fetal aneuploidy in samples of maternal plasma obtained in the first trimester of pregnancy. We demonstrated that MINK outperforms two contemporary methods and requires significantly fewer input sequencing reads. We conclude that MINK can be used in the clinical setting for cost-effective and accurate non-invasive prenatal aneuploidy detection.

## Supporting information

S1 Table Summary of Clinical and Demographic Characteristics of Study Population. Note that “amnio” = amniocentesis, “CVS” = chorionic villus sampling, “PN” = karyotype determined postnatally following a blood draw, “Path” = karyotype performed after elective or spontaneous termination. “Used as ref” indicates that the sample was used as a reference sample (see text).
(XLSX)

S1 Fig Box plot of p values of the MINK tests for chromosome 13 of the 63 trisomy samples (73 libraries) against the reference libraries. A library is colored in red if it is trisomy in the corresponding chromosome. A library is reported as trisomy if the median of the p values is less than or equal to 0.05. It is reported normal if the median p value is greater than or equal to 0.1. If the median p value is between 0.05 and 0.1, it is considered ambiguous and requires further investigation.
(PDF)

S2 Fig Box plot of p values of the MINK tests for chromosome 14 of the 63 trisomy samples (73 libraries) against the reference libraries. A library is colored in red if it is trisomy in the corresponding chromosome. A library is reported as trisomy if the median of the p values is less than or equal to 0.05. It is reported normal if the median p value is greater than or equal to 0.1. If the median p value is between 0.05 and 0.1, it is considered ambiguous and requires further investigation.
(PDF)

S3 Fig Box plot of p values of the MINK tests for chromosome 18 of the 63 trisomy samples (73 libraries) against the reference libraries. A library is colored in red if it is trisomy in the corresponding chromosome. A library is reported as trisomy if the median of the p values is less than or equal to 0.05. It is reported normal if the median p value is greater than or equal to 0.1. If the median p value is between 0.05 and 0.1, it is considered ambiguous and requires further investigation.
(PDF)

S4 Fig Box plot of p values of the MINK tests for chromosome 21 of the 63 trisomy samples (73 libraries) against the reference libraries. A library is colored in red if it is trisomy in the corresponding chromosome. A library is reported as trisomy if the median of the p values is less than or equal to 0.05. It is reported normal if the median p value is greater than or equal to 0.1. If the median p value is between 0.05 and 0.1, it is considered ambiguous and requires further investigation.
(PDF)

S5 Fig Box plots of p values of the MINK tests for chromosome 13 of the 355 normal samples (377 libraries) against the reference libraries. A library is reported as trisomy if the median of the p values is less than or equal to 0.05. It is reported normal if the median p value is greater than or equal to 0.1. If the median p value is between 0.05 and 0.1, it is considered ambiguous and requires further investigation.
(PDF)

S6 Fig Box plots of p values of the MINK tests for chromosome 14 of the 355 normal samples (377 libraries) against the reference libraries. A library is reported as trisomy if the median of the p values is less than or equal to 0.05. It is reported normal if the median p value is greater than or equal to 0.1. If the median p value is between 0.05 and 0.1, it is considered ambiguous and requires further investigation.
(PDF)

S7 Fig Box plots of p values of the MINK tests for chromosome 18 of the 355 normal samples (377 libraries) against the reference libraries. A library is reported as trisomy if the median of the p values is less than or equal to 0.05. It is reported normal if the median p value is greater than or equal to 0.1. If the median p value is between 0.05 and 0.1, it is considered ambiguous and requires further investigation.
(PDF)

S8 Fig Box plots of p values of the MINK tests for chromosome 21 of the 355 normal samples (377 libraries) against the reference libraries. A library is reported as trisomy if the median of the p values is less than or equal to 0.05. It is reported normal if the median p value is greater than or equal to 0.1. If the median p value is between 0.05 and 0.1, it is considered ambiguous and requires further investigation.
(PDF)
