# Supplementary material for: Comparative evaluation of the Minimally-Invasive Karyotyping (MINK) algorithm for non-invasive prenatal testing
Source: PLoS One. 2017 Mar 17;12(3):e0171882. doi: 10.1371/journal.pone.0171882 (PMC5356998; doi:10.1371/journal.pone.0171882)

S3 Fig. Box plots of p values of the MINK tests for chromosome 18 of the 63 trisomy samples (73 libraries) against the reference libraries. A library is colored in red if it is trisomy in the corresponding chromosome. A library is reported as trisomy if the median of the p values is less than or equal to 0.05. It is reported normal if the median p value is greater than or equal to 0.1. If the median p value is between 0.05 and 0.1, it is considered ambiguous and requires further investigation.

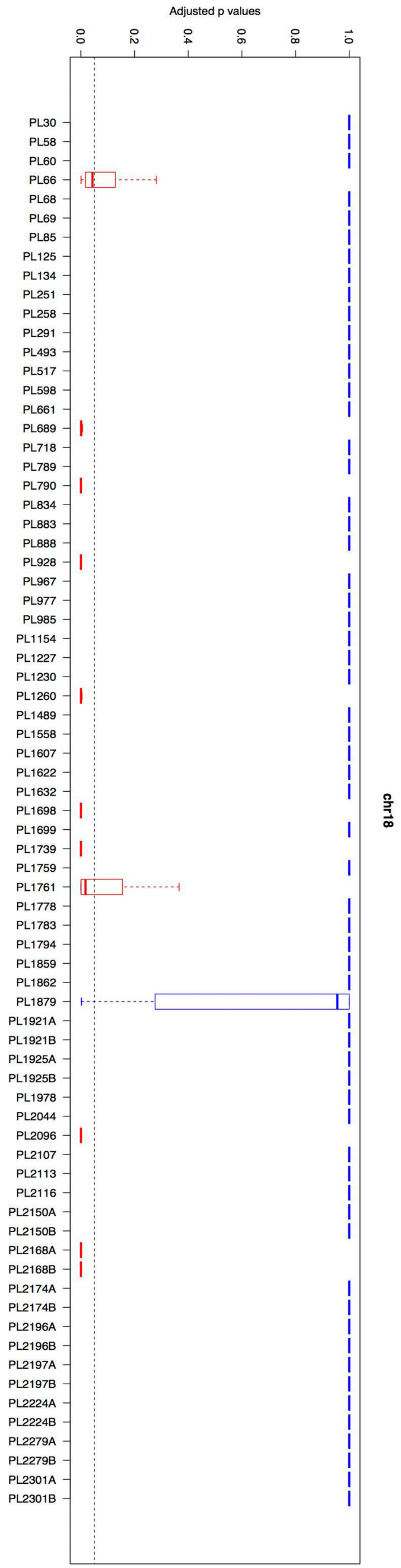

Supplement: S3 Fig — A library is colored in red if it is trisomy in the corresponding chromosome. A library is reported as trisomy if the median of the p values is less than or equal to 0.05. It is reported normal if the median p value is greater than or equal to 0.1. If the median p value is between 0.05 and 0.1, it is considered ambiguous and requires further investigation. (PDF) [file pone.0171882.s004.pdf]
